# Gelsolin Amyloidogenesis Is Effectively Modulated by Curcumin and Emetine Conjugated PLGA Nanoparticles

**DOI:** 10.1371/journal.pone.0127011

**Published:** 2015-05-21

**Authors:** Ankit Srivastava, Prabha Arya, Surbhi Goel, Bishwajit Kundu, Prashant Mishra, Ashish Fnu

**Affiliations:** 1 Kusuma School of Biological Sciences, IIT Delhi, New Delhi, India; 2 Department of Biochemical Engineering and Biotechnology, IIT Delhi, New Delhi, India; 3 CSIR Institute of Microbial Technology, Chandigarh 160036, India; Aligarh Muslim University, INDIA

## Abstract

Small molecule based therapeutic intervention of amyloids has been limited by their low solubility and poor pharmacokinetic characteristics. We report here, the use of water soluble poly lactic-co-glycolic acid (PLGA)-encapsulated curcumin and emetine nanoparticles (Cm-NPs and Em-NPs, respectively), as potential modulators of gelsolin amyloidogenesis. Using the amyloid-specific dye Thioflavin T (ThT) as an indicator along with electron microscopic imaging we show that the presence of Cm-NPs augmented amyloid formation in gelsolin by skipping the pre-fibrillar assemblies, while Em-NPs induced non-fibrillar aggregates. These two types of aggregates differed in their morphologies, surface hydrophobicity and secondary structural signatures, confirming that they followed distinct pathways. In spite of differences, both these aggregates displayed reduced toxicity against SH-SY5Y human neuroblastoma cells as compared to control gelsolin amyloids. We conclude that the cytotoxicity of gelsolin amyloids can be reduced by either stalling or accelerating its fibrillation process. In addition, Cm-NPs increased the fibrillar bulk while Em-NPs defibrillated the pre-formed gelsolin amyloids. Moreover, amyloid modulation happened at a much lower concentration and at a faster rate by the PLGA encapsulated compounds as compared to their free forms. Thus, besides improving pharmacokinetic and biocompatible properties of curcumin and emetine, PLGA conjugation elevates the therapeutic potential of both small molecules against amyloid fibrillation and toxicity.

## Introduction

Pathogenic manifestations in several neurodegenerative and systemic diseases including Alzheimer’s, Parkinson’s, Huntington’s, type II diabetes and prion diseases have been mainly attributed to fibrillar protein aggregates called amyloids [1–4]. Amyloid formation is the resultant of perturbed protein folding due to mutations, weakened ubiquitin-proteasome machinery, oxidative stress and metal imbalance [5, 6]. The current pharmacological intervention of amyloid diseases is limited owing to lack of information on the mechanism and the actual toxic species involved. Several non-specific chaperones and small molecules have been shown to alter amyloid pathway in a variety of amyloidogenic proteins but their pharmacokinetic relevance is yet to be tested [7–11]. Nevertheless, the ability of small molecules to induce conformational changes in proteins at low concentrations, negligible immunogenicity and ease of in *vitro* and in *vivo* testing makes them a preferred choice as amyloid modulators [12–14].

**Fig 1:**
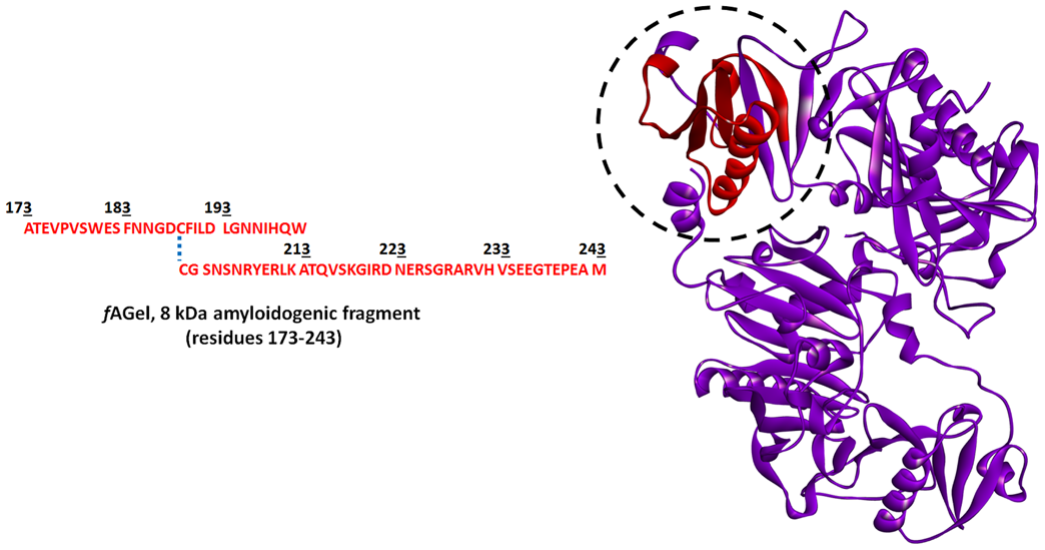
The crystal structure of human plasma gelsolin (PDB: 3FFN) showing the presence of 8 kDa amyloidogenic fragment (*f*AGel; highlighted in red). The amino acid sequence of *f*AGel is also shown.

Gelsolin is an actin-binding protein that remodels the actin cytoskeleton as well as scavenges actin fibrils released from injured tissues [15]. However, a mutation (D187N/Y) in gelsolin causes Familial Amyloidosis of Finnish Type (FAF), a disease characterized by amyloid deposits of an 8 kDa protein fragment (*f*AGel, residues 173–243, Fig 1). FAF is a systemic amyloidosis disease characterized by cranial lattice dystrophy which progresses with age and is associated with severe neuropathy [16]. Previously, we reported curcumin and emetine as potent binders of amyloidogenic stretch in gelsolin and modulate its amyloidogenic pathway [17]. However, a major drawback in the applicability of both curcumin and emetine in physiological systems is their poor solubility, high hydrophobicity and instability [18].

A practical way to overcome this drawback is the preparation of nanoparticles (NPs) that entail higher solubility and surface availability, along with improved stability [19–22]. Generally, an increase in solubility enhances bioavailability that delays rapid metabolism and systemic elimination [23]. Moreover, NPs can pass through the blood-brain barrier and due to high levels of surface free energies it shows increased adsorption [24–26]. PLGA (Poly (lactic-co-glycolic acid)) is formed by copolymerization of the cyclic dimers of glycolic acid and lactic acid. The ester linkage of PLGA undergoes hydrolysis in aqueous systems, releasing the monomers which are easily metabolized and removed from the body [27]. Moreover, PLGA nanoparticles with some surface modification having sizes below 300 nm have been shown to pass through the blood brain-barrier [28, 29].

Thus, owing to the biocompatible and biodegradable properties, PLGA has been approved by the Food and Drug Administration for usage in therapeutics [30]. Here we report that PLGA-loaded curcumin and emetine nanoparticles modulate the fibrillation of a disease-associated gelsolin amyloidogenic fragment (*f*AGel) in a much efficient way as compared to the non-encapsulated forms of the compounds.

## Materials and Methods

### Chemicals and reagents

Curcumin, Poly (D,L-lactide-co-glycolide) (PLGA), MW (40,000–75,000) co-polymer ratio (50:50), Poly (vinyl alcohol) (PVA), average MW (30,000–70,000), viscosity 4–6 cP (4% aqueous solution, 20°C), Dimethyl sulfoxide (DMSO) and Dichloro-methane (DCM) were purchased from Sigma-Aldrich, USA. Emetine was purchased from Calbiochem, India. The cell culture medium (RPMI 1640), penicillin-streptomycin and fetal bovine serum (FBS) were obtained from Gibco BRL (Life technology, Paisley, Scotland). All other chemicals of analytical grade were purchased from Merck (India).

### Cloning, expression and purification of Protein

The 213 base pair DNA sequence coding for the 8 kDa (Ala 173-Met 243) amyloidogenic fragment (*f*AGel) was amplified by polymerase chain reaction (PCR) using human plasma gelsolin cDNA template (Gene bank accession number AK295572.1). The following primer pairs CCGTCCATGGGCCACCGAGGTACCTGTG and CCGTCTCGAGCATCGCCTCGGGCTCAGTG were used. The amplified cDNA sequence was inserted in the pGEX-6P2 vector (GE Healthcare, USA) downstream of a GST coding sequence (*NcoI* and *XhoI*). This GST- *f*AGel fusion clone was transformed into *E*. *coli* BL21 (DE3) cells (Promega, USA) for protein expression using the standard IPTG induction protocol. The cells were grown in Luria broth (LB), containing 100 μM ampicillin at 37°C shaking, until an OD_600nm_ of 0.6 was reached. At this stage, the cells were induced by 1 mM IPTG and further grown for 4–5 h before harvesting by centrifugation (3000 rcf). The cell pellet obtained was re-suspended in buffer A (50 mM Tris, 100 mM NaCl; pH 8.0) containing 1X protease inhibitor cocktail (Sigma Inc., USA) and incubated for 30 minutes on ice before sonicating for 15 cycles (2 min ON/1 min OFF) at 40 W (output 6; 50% duty cycle) in ice in a Q700 sonicator (QSonica, USA). The sonicated cell suspension was centrifuged at 12000 rcf for 1 h to remove cell debris. The resulting cell lysate was mixed with glutathione sepharose resin (GST resin, GE Healthcare, USA) pre-equilibrated with buffer A and left for incubation at 4°C for 1 h to allow the protein to bind. After the incubation period, the mixture was loaded onto a column (column volume 5 ml) in batch mode and the flow through was collected. After washing the column with buffer A (5 column volumes), protein was eluted with pre-chilled elution buffer (50 mM Tris, 100 mM NaCl and 10 mM reduced glutathione; pH 8.0). Following this, the eluted fractions were collected and dialyzed extensively against buffer A at 4°C to remove glutathione. The purified GST-tagged protein was incubated overnight with precision protease (0.2 U per mg protein) at 4°C to remove the GST-tag. The cleavage was confirmed by SDS-PAGE (15%). The residual GST-tags were removed by loading the protein solution onto a GST column, pre-equilibrated with buffer A. A second step of purification involved 10 kDa centrifugal filters to remove any residual GST impurity. The obtained 8 kDa *f*AGel fragment was confirmed on 20% SDS PAGE, was concentrated, lyophilized and stored at -80°C.

### Preparation of Nanoparticles

Emetine and curcumin were encapsulated in PLGA by water/oil/water and oil/water method, respectively [31, 32]. A 10 mM stock solution of both curcumin and emetine were made in DMSO. Initially, 90 mg of PLGA was added to 2 ml of DCM along with 1 ml stock solution of curcumin and vortexed to prepare the primary emulsion. In the case of emetine, stock solution of emetine was added in PLGA-DCM solution step by step (100 μl at a time) and vortexed, followed by addition into the 2% PVA solution (12 ml) and mixed. Both kinds of suspensions (~15 ml each) were sonicated (output 6; 40% duty cycle) for 3 minutes for secondary emulsion and were left on stirrer overnight to evaporate the DCM by desiccation. Following three washing steps by centrifugation (45,000 rcf at 4°C, 20 minutes), pellets were resuspended in miliQ water. Larger aggregates were removed by centrifugation and supernatant was lyophilized.

### Particle size and zeta potential of Nanoparticles

Zeta potential and size of the curcumin and emetine nanoparticles was determined using Zetasizer Nano ZS 3000 (Malvern Instruments, UK) as described earlier [33]. For particle size, scans were taken in duplicates with a run of 10 seconds correlation time. Intensity of distribution (%) of particles was plotted against hydrodynamic radius (nm). For zeta potential, measurements of blank and NPs in milliQ water were taken using default parameters of dielectric constant, refractive index, and viscosity. All measurements were done in triplicates at 25°C. Total counts of particles were plotted against Zeta potential (mV). The built in Zetasizer software 6.01 was used for all analysis.

### Determination of entrapment efficiency (EE)

Curcumin and emetine content in the NPs were determined using UV-spectrophotometric analysis. A standard curve was made by taking absorbance of the compounds with a range of known concentrations. The absorbance was taken at 425 nm and 236 nm for curcumin and emetine, respectively [34, 35]. The test samples were prepared by dissolving the NPs in 0.1 N NaOH and heating at 50°C for an hour to ensure complete release of the compounds. The absorbance of resulting supernatant was taken at respective wavelengths and concentration was calculated from the standard curve. All absorbance values were recorded in triplicates and the entrapment efficiency was calculated using the following equation, where the fraction is defined as actual loading (μg/mg) of compounds [36]

$$Entrapment\;efficiency\;=\;\frac{[compound]\;total-[compound]\;free}{[compound]\;total}x\;100$$

### FTIR spectroscopy

FTIR analysis of NPs was performed to confirm the functional groups of the compounds. For this, dried PLGA-NPs, Cm-NPs and Em-NPs were mixed with spectroscopic grade KBr (Sigma-Aldrich), pressed into a pellet and the spectrum taken immediately. All spectra were recorded using a Nicolet 6700 FTIR spectrometer (Thermo Scientific, USA) with an inbuilt (MCT A) photo detector purged with dry air. For characterizing the secondary structural signatures of the aggregates, ATR-FTIR was used. For this, 24 h incubated aggregates were centrifuged and resuspended in 50 μl of 0.1 M acetate buffer, pH 5.0. A drop of aggregate solution was applied on the germanium crystal of the horizontal ATR sampling accessory. In all cases data was acquired at a resolution of 4 cm^-1^ and 128 scans were averaged per sample. The background (buffer/KBr) was subtracted. The amide I region between 1700 cm^-1^ to 1600 cm^-1^ was analysed for characteristic changes in the polypeptide chain of aggregated species based on previously described peak assignments [37, 38]. The OMNIC 32 software was used to record and process the spectra.

### Aggregation assay

For aggregation reactions, samples containing *f*AGel alone (25 μM, 2 mg/ml) or with varying concentrations of NPs (0.01, 0.05, 0.1 mg/ml) were made in 50 mM acetate buffer (pH 4.0) containing 150 mM NaCl, 0.02% NaN_3_ and incubated at 37°C, without agitation [39]. For defibrillation assay, *f*AGel fibrils were grown up to 4 days and then NPs were added (0.05, 0.1 mg/ml) into the solution and incubated at 37°C, without agitation.

### Dye binding assays

Aliquots withdrawn at different time points and were diluted into buffer (50 mM acetate buffer, pH 5.0) containing 10 μM ThT and adjusted to a final volume of 100 μl. Fluorescence spectra of these samples were recorded by using LS 55 fluorescence spectrometer (Perkin Elmer, MA, USA), keeping the excitation and emission wavelengths at 440 nm and 485 nm, respectively. The averaged fluorescence data points from triplicate reactions for each concentration were fitted to sigmoidal curve using the following equation in Origin 8.0.

$$F\;=\;\frac{A2-A1}{[1+exp\frac{\left(t-t0.5\right)}{k}]\;}+\;A2$$

Here, A1 and A2 represent initial and final fluorescence intensities respectively, t0.5 is the time at half-maximum fluorescence and k is the rate constant. The lag phase was calculated as t0.5-2k.

For Nile red binding assay, aliquots were diluted with 50 mM acetate buffer, pH 5.0 containing 5 μM Nile red and adjusted to a final volume of 100 ul. Fluorescence emission spectra of triplicate samples were recorded from 550–700 nm after excitation at 530 nm. In all cases, the excitation and emission slit width were kept at 5 nm and 10 nm respectively.

### Transmission Electron Microscopy (TEM)

TEM images of aggregating samples were acquired using Tecnai transmission electron microscope (FEI, Eindhoven, The Netherlands) equipped with a bright field/dark field detector operating at 120 kV. The *f*AGel aggregates formed in presence or absence of Cm-NPs or Em-NPs obtained after one week of incubation were imaged. 2–3 μl of 5-fold diluted samples were placed on copper grid for 2 minutes. Following this, the samples were negatively stained using a 2% (w/v) uranyl acetate solution and washed with milliQ water, air dried and imaged. The imaging was done at a magnification of 86,000X with an electron dose of 20–30 e/Å^2^.

### Atomic Force Microscopy (AFM)

Aqueous suspension of NPs or three fold diluted aggregating samples (~5 ul) were applied on freshly peeled mica surface. After 5–10 minutes, samples were washed and dried under nitrogen flush. Imaging was done using Bioscope Catalyst AFM (Bruker, USA) in tapping mode. Images were processed using nanoscope analysis v.1.4.

### Scanning Electron Microscopy (SEM)

Aqueous suspensions of NPs were spread evenly over a slab, dried and gold-coated. Similarly, *f*AGel aggregating samples incubated for 2 weeks, with or without NPs were also prepared. Each sample was visualized using scanning electron microscope (Evo50, Zeiss, UK) operating at 5 keV.

### Cell culture and toxicity assessment

The human SH-SY5Y cell line (ATCC, USA) was maintained in DMEM supplemented with 10% fetal bovine serum (Gibco, UK) and incubated at 37°C under 5% CO_2_. About 6x10^4^ viable cells were seeded in 96 well plates with overnight media replacement. The *f*AGel aggregates formed after 24 hour of incubation either alone or in the presence of different concentrations of NPs were pelleted and resuspended in PBS (NaCl 137 mM, KCl 2.7 mM, Na_2_HPO_4_ 10 mM, KH_2_PO_4_ 1.8 mM). These aggregates (25 μl) were added to the cells and incubated for 24 hours. Following this, the medium was replaced with the MTT containing medium (1 mg/ml) and the cells were further incubated for 4 hours at 37°C. After incubation, medium was removed and cells were diluted in 200 μl of DMSO. The relative formazan formation in each well was measured by determination of absorbance at 570 nm in a microplate reader (MultiSkan, Thermo Scientific, USA). The resulting absorbance values were converted to percentage viability with respect to the untreated control cells. The differences between the sampling groups were checked for statistical significance using student's t-test.

## Results

### Preparation and characterization of nanoparticles

**Fig 2:**
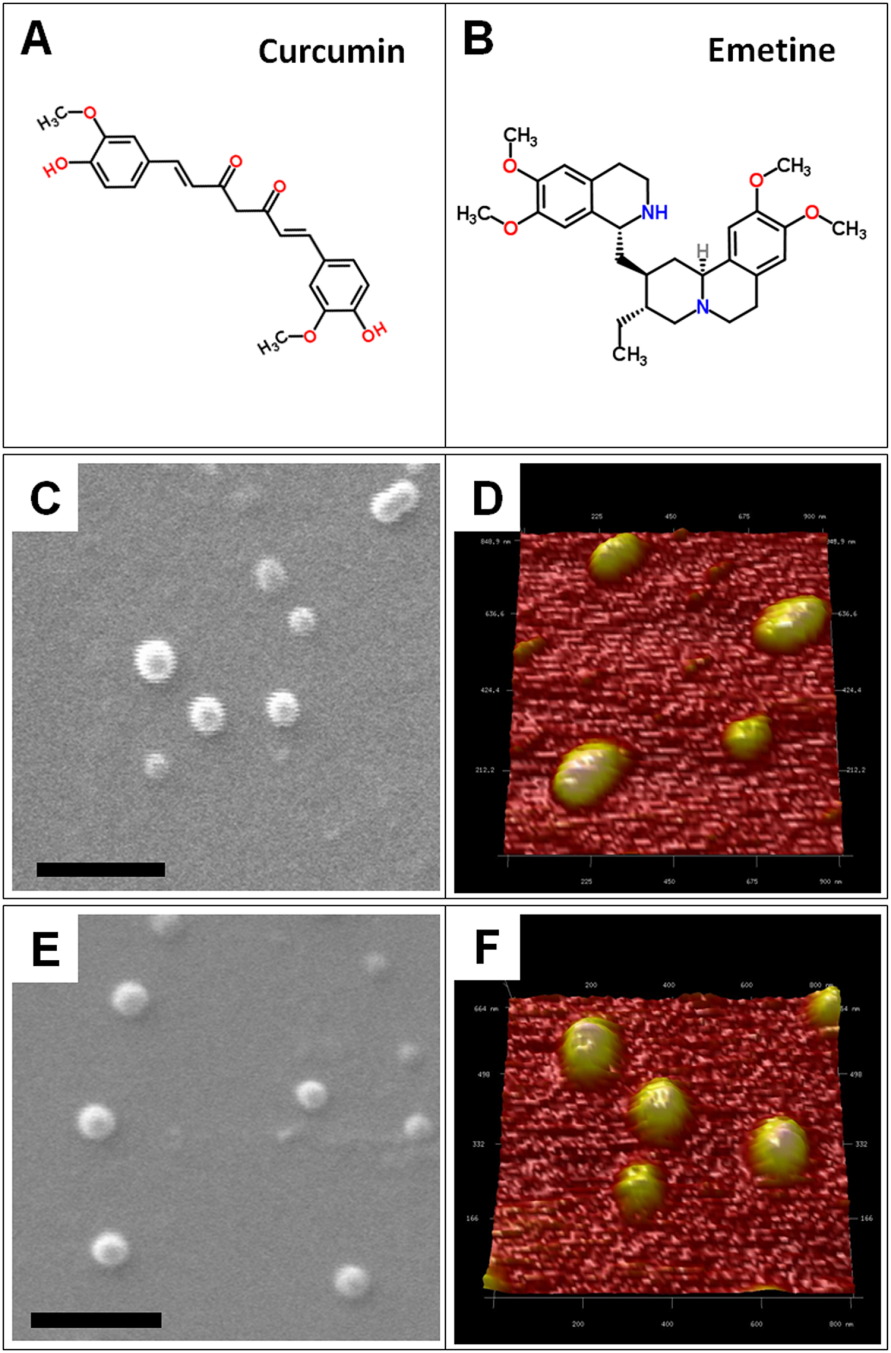
Chemical and morphological characteristics of nanoparticles. The chemical structure of curcumin (A) and emetine (B), SEM (C) and AFM (D) images of Cm-NPs, SEM (E) and AFM (F) images of Em-NPs. The scale bar in SEM images represents 1 μm.

**Fig 3:**
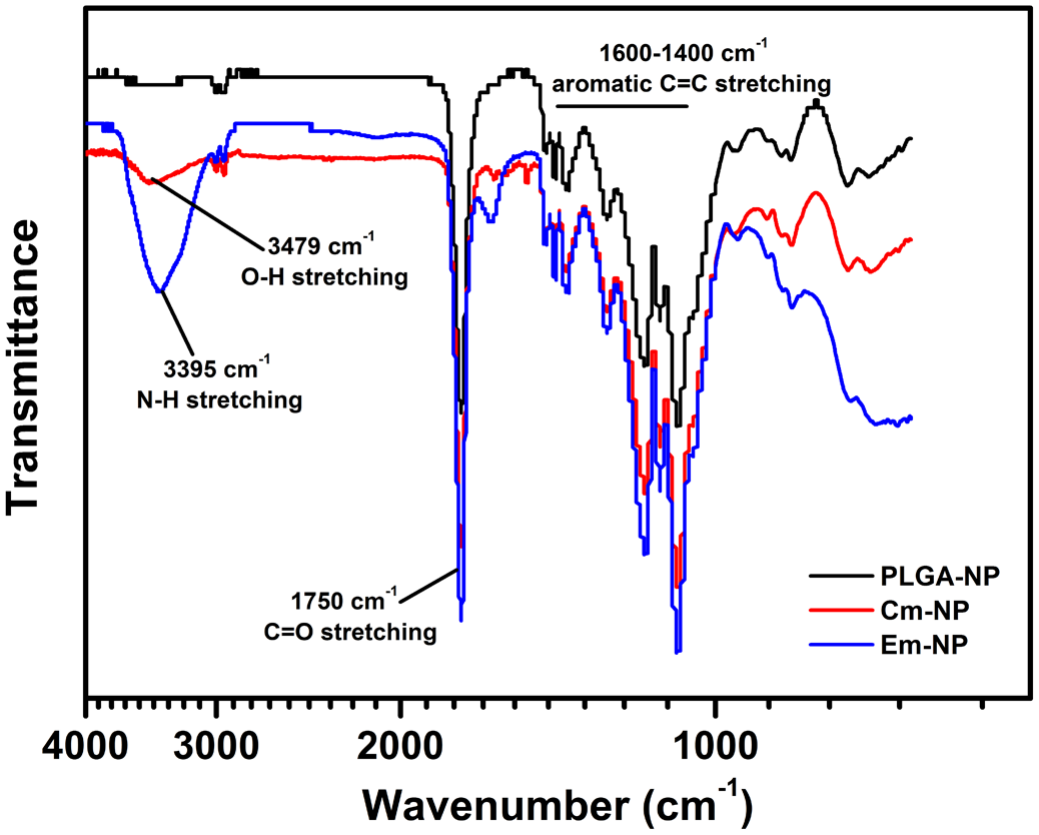
Fourier transformed infrared spectra of nanoparticles. FTIR spectra of Cm-NPs (red), Em-NPs (blue) and free PLGA-NPs (black) showing overlapping peaks of C = O stretching, corresponding to PLGA. Distinct—OH and—NH stretching peaks characteristic of curcumin and emetine, respectively are also shown.

The biological activity of both the compounds depend on their specific functional groups *viz*. phenol rings in curcumin and isoquinoline ring in emetine (Fig 2A and 2B). PLGA polymer was used for encapsulation of curcumin or emetine by oil in water and water in oil in water solvent evaporation method, respectively. The corresponding SEM and AFM analysis showed roughly spherical morphology of both the NPs (Fig 2C & 2D; 2E & 2F). The physico-chemical properties of the NPs are presented in Table 1. The nanoparticles showed narrow size distribution with an average diameter of 261.5±8 nm. To assess the surface charge, their average zeta potential was determined and was found as -30.8 mV, -37.6 mV and -35.9 mV for the Em-NPs, Cm-NPs the control PLGA NPs, respectively (S1 Fig). The FTIR spectra of NPs are shown in Fig 3. A characteristic C = O absorption band at 1750 cm^-1^ of PLGA was observed in all the three cases. In the Cm-NPs, additional peak of curcumin at 3379 cm^-1^ characteristic of O-H stretching was observed. For the Em-NPs, very sharp peak of isoquinoline at 3395 cm^-1^ characteristic of N-H stretching was observed. The aromatic signatures of C-C were found overlapping in all the samples. Based on actual loading of compounds, entrapment efficiency (EE) of Cm-NPs and Em-NPs was found ~19% and ~27%, respectively (Table 1).

**Table 1:** Physicochemical characteristics of nanoparticles.

Formation	Size (nm)	Polydispersity	Zeta Potential (mV)	Entrapment efficiency (%)	Actual Loading (μg/mg NP)
NPs	263.5±7.8	0.071±0.06	-35.9±1.5	-	-
Cm-NPs	261±2.4	0.039±0.08	-37.6±1.1	19	190
Em-NPs	260.1±8.1	0.085±0.05	-30.8±1.3	27	270

### *f*AGel exhibits nucleation-dependent aggregation

**Fig 4:**
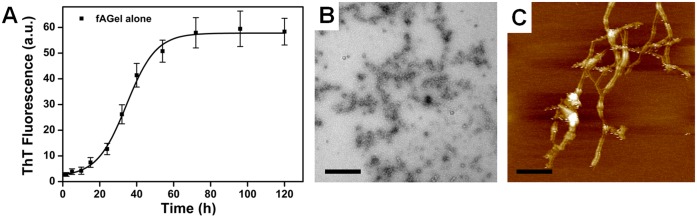
Kinetics and morphology of *f*AGel aggregation. (A) Amyloid formation kinetics of *f*AGel alone monitored by ThT fluorescence showing sigmoidal trend with 18 h lag phase. (B) TEM image of amyloids formed after 24 h incubation. Scale bar 500 nm. (C) AFM image taken after 60 h showing fibrillar aggregates of *f*AGel. Scale bar 200 nm. The *f*AGel concentration was 2 mg/ml.

The aggregation kinetics of *f*AGel at pH 4.0, 37°C was followed by monitoring the changes in Thioflavin T (ThT) fluorescence, a widely used fluorescent dye used for characterizing amyloid kinetics [40]. Evidently, the *f*AGel aggregation showed a time-dependent increase (k = 7.9 h^-1^) in the ThT fluorescence, followed a sigmoidal trace before saturating around 60 h (Fig 4A). A distinct lag phase (~18 h) was observed, suggesting that the polypeptide undergoes nucleation-dependent polymerization under these conditions, a phenomenon generally reported for other amyloidogenic polypeptides [41–43]. The TEM images taken after 24 h showed the presence of protofilaments and early fibrillar aggregates (Fig 4B). The corresponding AFM images taken after 60 h incubation showed the presence of mature fibrils of 8.6 nm average width and 5.2 nm average height having smooth morphologies (Fig 4C, S2 Fig).

### Testing the effect of NPs on *f*AGel aggregation

Initially, the kinetics of amyloid aggregation in *f*AGel was characterized by ThT fluorescence assay along with TEM and AFM imaging. The presence of protofilaments and mature fibrils at 24 h and 60 h respectively, during *f*AGel aggregation led us to choose these time points as benchmarks for structural characterization of *f*AGel aggregates formed in presence of NPs. For characterizing the effect of different concentrations of NPs on aggregation kinetics of *f*AGel, ThT assay of aliquots withdrawn at different time points was done. Following this, samples were characterized for morphological differences (24 h and 60 h) using TEM and AFM imaging. The surface hydrophobicity and secondary structural changes were compared only for the 24 h samples since they exhibited highest degree of morphological differences. Since we were also interested in investigating the effect of NPs on amyloid toxicity, pre-fibrillar aggregates of *f*AGel observed at the end of 24 h were chosen for relative assessment of toxicity of aggregates formed in presence of NPs at the same time point. In addition, for checking the effect of NPs on pre-formed amyloids, *f*AGel was allowed to aggregate for an extended period of time (4 days) to ascertain that the sample only contains mature fibrils. Following this, the effect of NPs was then substantiated using ThT assay in a time-wise manner and TEM imaging. The details are described in the methods section.

### Cm-NPs augment and Em-NPs arrest *f*AGel fibrillation

**Fig 5:**
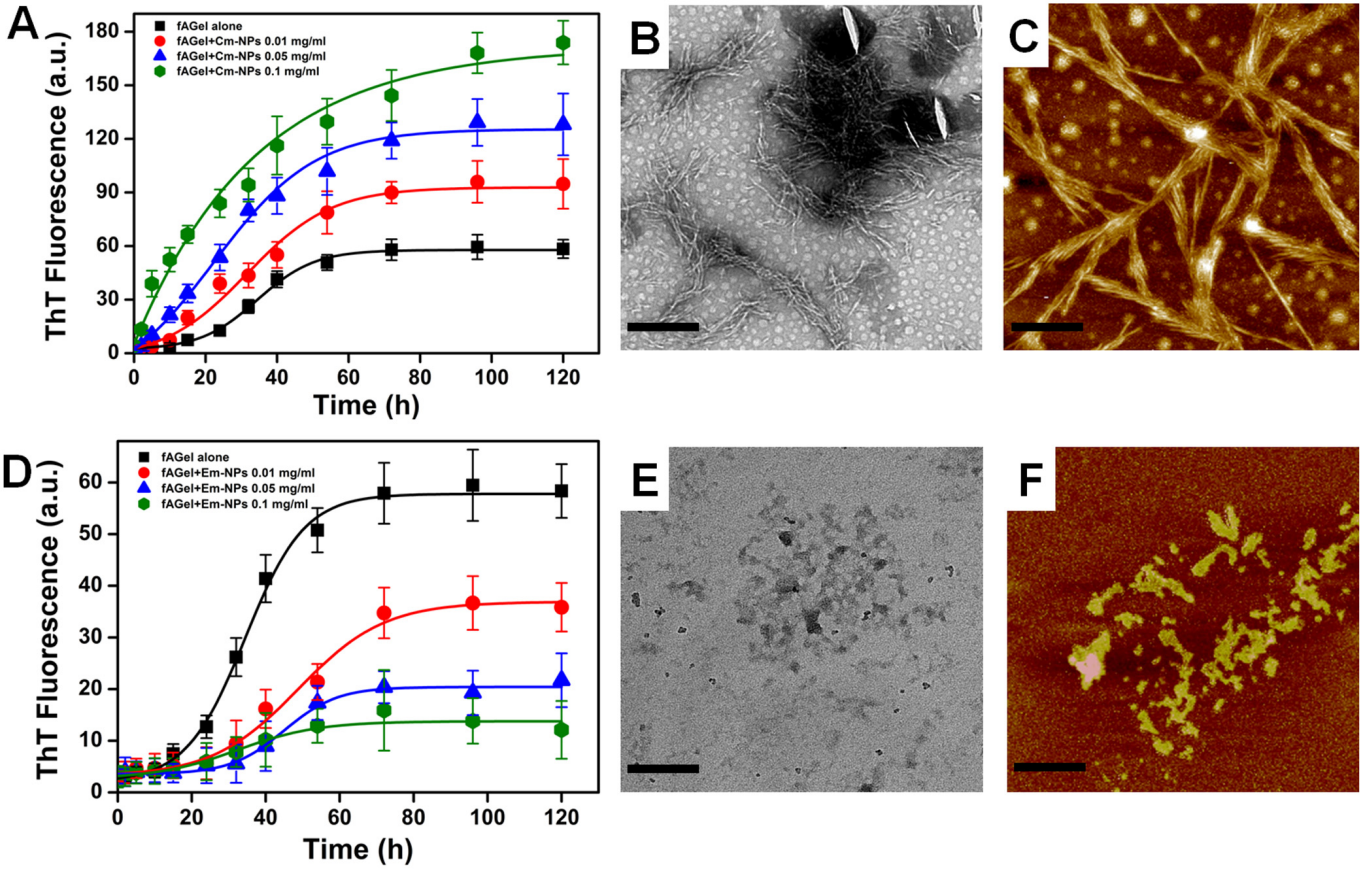
Kinetics and morphologies of NP- incubated *f*AGel aggregates. *f*AGel aggregation kinetics monitored by ThT fluorescence in the absence (black trace) or in the presence (coloured traces) of different concentrations of NPs. (A) Acceleration of aggregation in the presence of increasing concentrations of Cm-NPs. The corresponding TEM (B) and AFM (C) images are shown. (D) Suppression of aggregation in the presence of increasing concentrations of Em-NPs with corresponding TEM (E) and AFM (F) images. In each case the TEM and the AFM images were taken after 24 h and 60 h of *f*AGel incubation with 0.1 mg/ml of respective NPs. Scale bars represent 500 nm for TEM and 200 nm for AFM images.

We tested the effect of Cm-NPs and Em-NPs at varying concentrations (0.01, 0.05, 0.1 mg/ml) on *f*AGel aggregation kinetics using ThT fluorescence and compared it with control. In contrast to the nucleation polymerization kinetics seen in *f*AGel alone, the presence of Cm-NPs augmented the fibrillation process. Both the ThT fluorescence maxima (F_max_) and the apparent rate of polymerization increased by more than 150% (F_max_ = 92.8; k = 11.8 h^-1^) in presence of minimum Cm-NP concentration (0.01 mg/ml) (Fig 5A). On increasing the concentration of Cm-NPs, the aggregation kinetics increased exponentially with no apparent lag phase. A strikingly high ThT fluorescence maxima (>300% at 0.1 mg/ml Cm-NP concentration) indicated the presence of large amount of fibrillar aggregates. This was confirmed by TEM micrographs of Cm-NP samples that showed dense fibrillation with individual fibrils sticking and interweaving together (Fig 5B). Further, on analysing the AFM images of 60 h incubated samples, laterally stacked fibrils in bunches of 8–10 individual fibers were observed (Fig 5C). The fibrillar bunches were having an average width and height of 33.3 nm and 27.2 nm respectively indicating relatively high fibrillar bulk (S3 Fig). One possible explanation could be the availability of larger number of soluble curcumin moieties in the form of NPs, resulting into higher occurrence of stacking interactions. This corroborates well with our recent report of curcumin binding to the core amyloidic stretch of gelsolin (AGel, 182–192 residue) through tri-planar π-π stacking interactions with PHE 183 and PHE 189 residues [17]. This also supports previous reports where aromatic interactions were shown to affect amyloid aggregation [44–46].

Contrary to this, in the presence of Em-NPs, the *f*AGel aggregation kinetics de-escalated in a dose dependent manner. At increasing concentrations of Em-NPs, the ThT fluorescence subdued significantly, with less than 25% fluorescence observed at the highest NP concentration of 0.1 mg/ml (Fig 5D). With a gradual increase in the lag phase with increasing concentrations (~26 h at 0.01 mg/ml and ~35 h at 0.05 mg/ml), the sigmoidal kinetics of *f*AGel aggregation was lost and subsequently arrested at the highest Em-NP concentration. In these samples, irregular shaped aggregates were observed under TEM, suggesting the inhibition of amyloid fibrillation in the presence of Em-NPs (Fig 5E). In this case, nano-encapsulation plausibly increased the number of emetine molecules accessing the *f*AGel polypeptide, in turn accelerating the steric blockage on *f*AGel aggregation by the bulky isoquinoline rings [17]. These observations were also ascertained by the AFM images of 60 h incubated samples, showing randomly shaped structures devoid of any distinct morphology (Fig 5F). The width and height of these unstructured aggregates were unevenly distributed, indicating low structural content (S6 Fig).

### Cm-NPs and Em-NPs alter hydrophobic exposure and secondary structure

**Fig 6:**
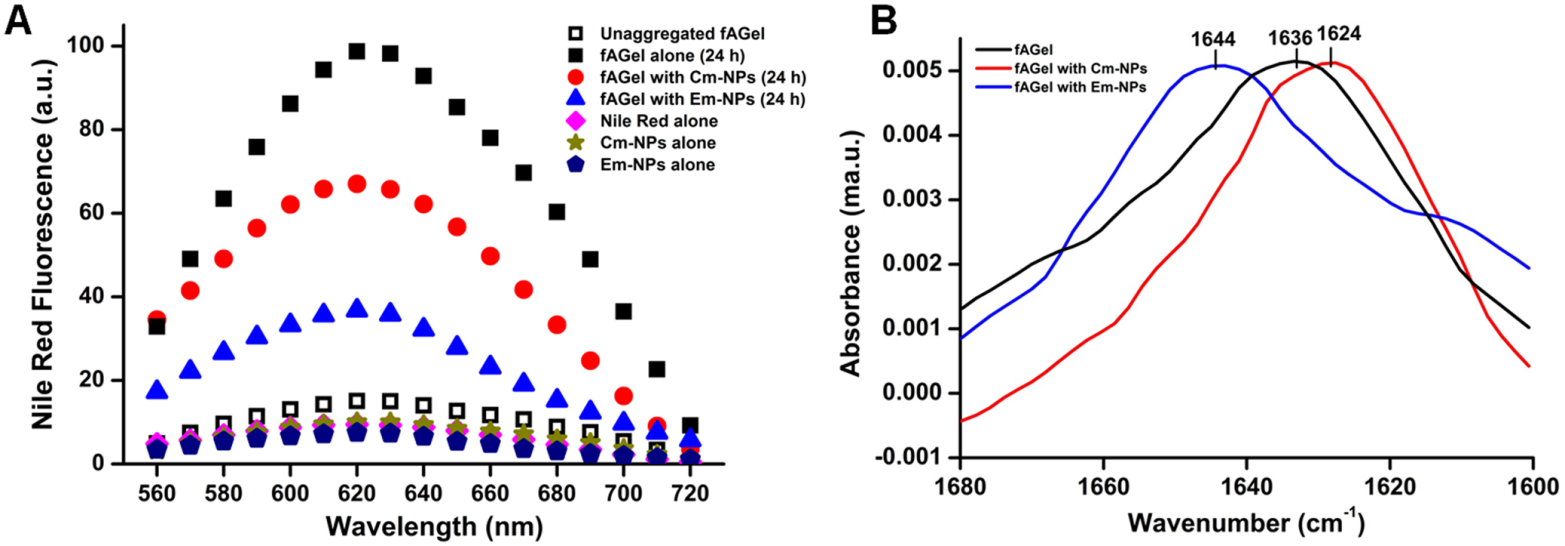
Hydrophobicity and secondary structure assessment of the aggregates. (A) Nile Red fluorescence of *f*AGel alone (black squares), with Cm-NPs (red circles) and Em-NPs (blue triangles). The fluorescence spectra of controls are also shown in colour. (B) ATR-FTIR spectra of aggregating samples of *f*AGel alone (black), with Cm-NPs (red) and with Em-NPs (blue) are compared. In each case the samples were taken after 24 h of incubation with NPs at 0.1 mg/ml concentration. A characteristic 1644 cm^-1^ peak for Em-NP sample corresponds to majorly unstructured aggregates.

The amyloid assembly progresses through interaction of hydrophobic surfaces often associated with secondary structural changes. Since, the extent of hydrophobic exposure is associated with the toxicity of the amyloid aggregates, we were interested in evaluating the same under the influence of NPs [47, 48]. For this, we employed Nile Red (NR) fluorescence assay (previously used for assessing hydrophobicity) on the 24 h incubated *f*AGel samples (both with and without NPs), since they exhibited highest degree of morphological differences in TEM and AFM images [49]. Evidently, Cm-NP treatment resulted in reduced NR binding that indicates reduction in surface/volume ratio in Cm-NP samples containing mature fibrils as compared to control that correspond to early/premature aggregates (Fig 6A). On the other hand, Em-NPs caused dramatically reduced NR fluorescence as compared to control *f*AGel aggregates confirming the unstructured and random aggregate species seen in TEM and AFM images. To further associate these alterations in the hydrophobic surfaces with any secondary structural changes under the influence of NPs, we performed ATR-FTIR analysis of these aggregates.

ATR-FTIR is a sensitive spectroscopic probe to evaluate secondary structural contents in proteins. The FTIR spectrum of *f*AGel showed a major peak at 1636 cm^-1^ which is attributed to the presence of β-sheets rich aggregates (Fig 6B). On the other hand, the Cm-NP influenced aggregates showed a major shift in peak position at 1624 cm^-1^, indicating the presence of fibrillar axis composed of extensive inter-molecular cross-β sheets [37, 50]. However, the presence of major peak at 1644 cm^-1^ in Em-NP samples was suggestive of random and unstructured aggregate species that validates the observed reduction in hydrophobic surface.

### Cm-NPs and Em-NPs reduce toxic assembly of *f*AGel amyloids

**Fig 7:**
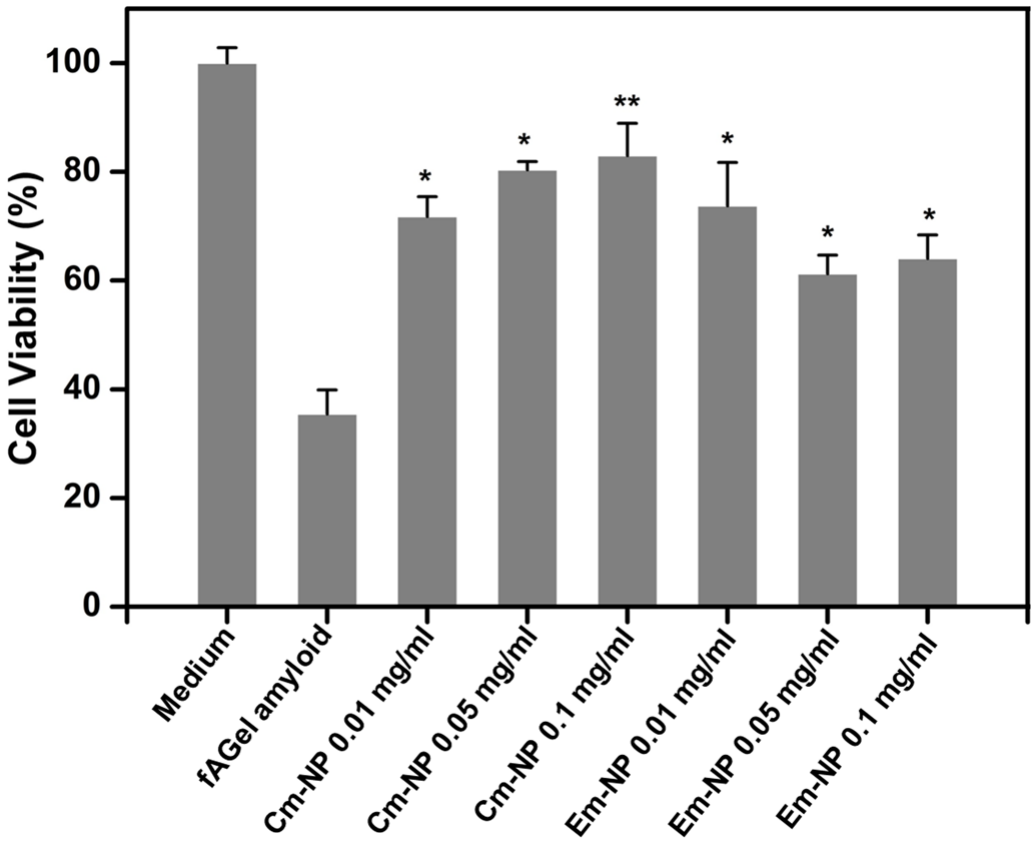
Cytotoxicity profile of aggregates. Toxicity assessment of *f*AGel amyloids and aggregates formed at different concentrations of Cm-NPs or Em-NPs on SH-SY5Y cells, represented as percentage of untreated control. In each case the 24 h incubated aggregates were used.

The fibrillar and non-fibrillar aggregates formed in the presence of Cm-NPs and Em-NPs were tested for their toxic effects on the human neuroblastoma cells (SH-SY5Y). Since early proto- or pre-fibrillar aggregates are shown as the toxic amyloid species, we chose the 24 h incubated samples showing significant morphological differences [51]. The *f*AGel aggregates alone displayed high cellular toxicity reducing the cell viability to less than 40% after 24 h of incubation (Fig 7). Interestingly, while both the Cm- and Em-NP-induced aggregates displayed reduced cellular toxicity, the viability showed an upward trend in the case of Cm-NPs in a dose dependent manner. In the case of Em-NPs however, there was a loss of cell viability at higher concentrations which is probably due to the inhibitory effect of emetine on protein synthesis [52].

### Cm-NPs increase fibrillar bulk and Em-NPs defibrillate amyloid fibers

**Fig 8:**
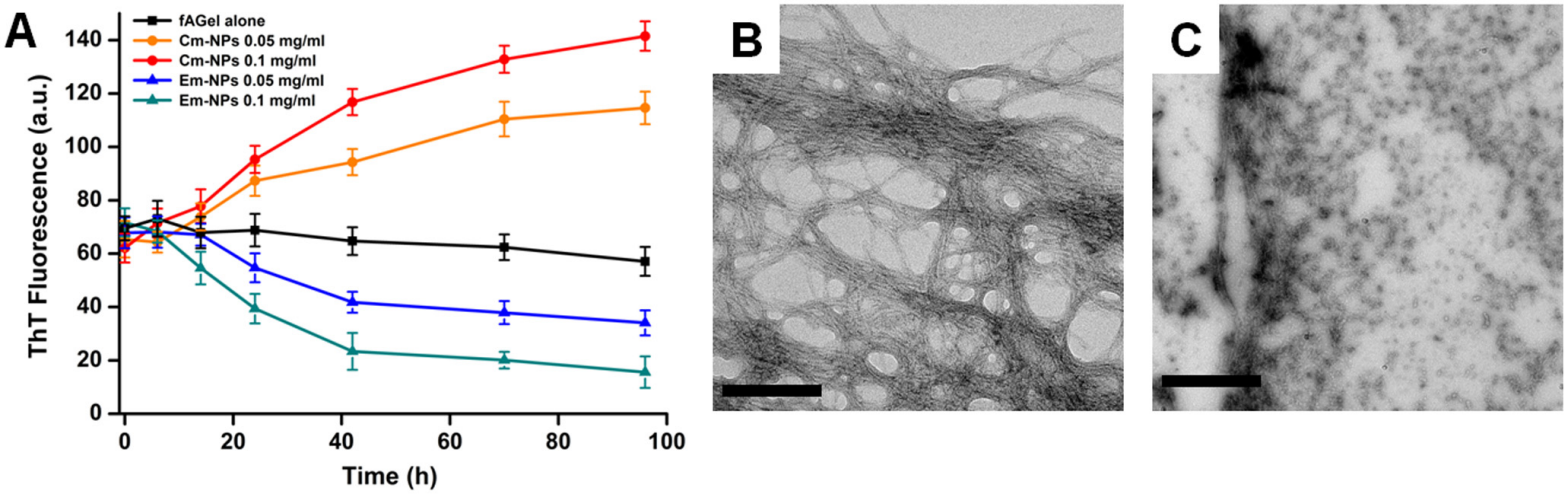
Effect of nanoparticles on pre-formed amyloids. (A) Time-dependent ThT fluorescence showing effect of Cm-NPs (solid circles) and Em-NPs (solid triangles) on pre-formed amyloids of *f*AGel (control, black solid squares). The electron micrographs of aggregates after incubation at 37°C for a week in the presence of 0.1 mg/ml of Cm-NPs (B) and Em-NPs (C). Scale bars represent 500 nm in each case.

We further checked whether both the NPs have any effect on pre-formed amyloids of *f*AGel. For this, we incubated *f*AGel amyloid fibrils with Cm-NPs or Em-NPs at 0.05 mg/ml and 0.1 mg/ml, and monitored the ThT response in a time-wise manner. We observed that at both the concentrations of Cm-NPs, a time dependent increase in ThT fluorescence was noted (Fig 8A). To visualize the associated morphological changes, we obtained TEM micrographs of the 7 day incubated samples which showed dense fibrillation and increase in fibril bulk (Fig 8B) [17]. The resulting higher order aggregates were thicker (~25–30 nm), rich in β-sheets and exhibited enhanced ThT response (>80% increase). In contrast, *f*AGel amyloids incubated with Em-NPs exhibited a decline in ThT response with time (~65% decline) and showed smaller, detached fragments with reminiscent of fibrillar axis (Fig 8C).

## Discussion

The present study describes the synthesis of PLGA nanoparticles encapsulating curcumin and emetine molecules, and their differential effects on gelsolin amyloids. Although small molecule inhibitors have been promoted as potential anti-amyloidogenic agents, their applicability falls way below the standard guidelines defined for drug candidates [53]. We had previously reported the interaction of curcumin and emetine, on the amyloidogenic propensity of a gelsolin-derived core amyloidogenic peptide AGel [17]. However, the poor solubility of the compounds and insufficient information about the actual disease-associated gelsolin fragment (*f*AGel), led us to initiate this investigation.

**Fig 9:**
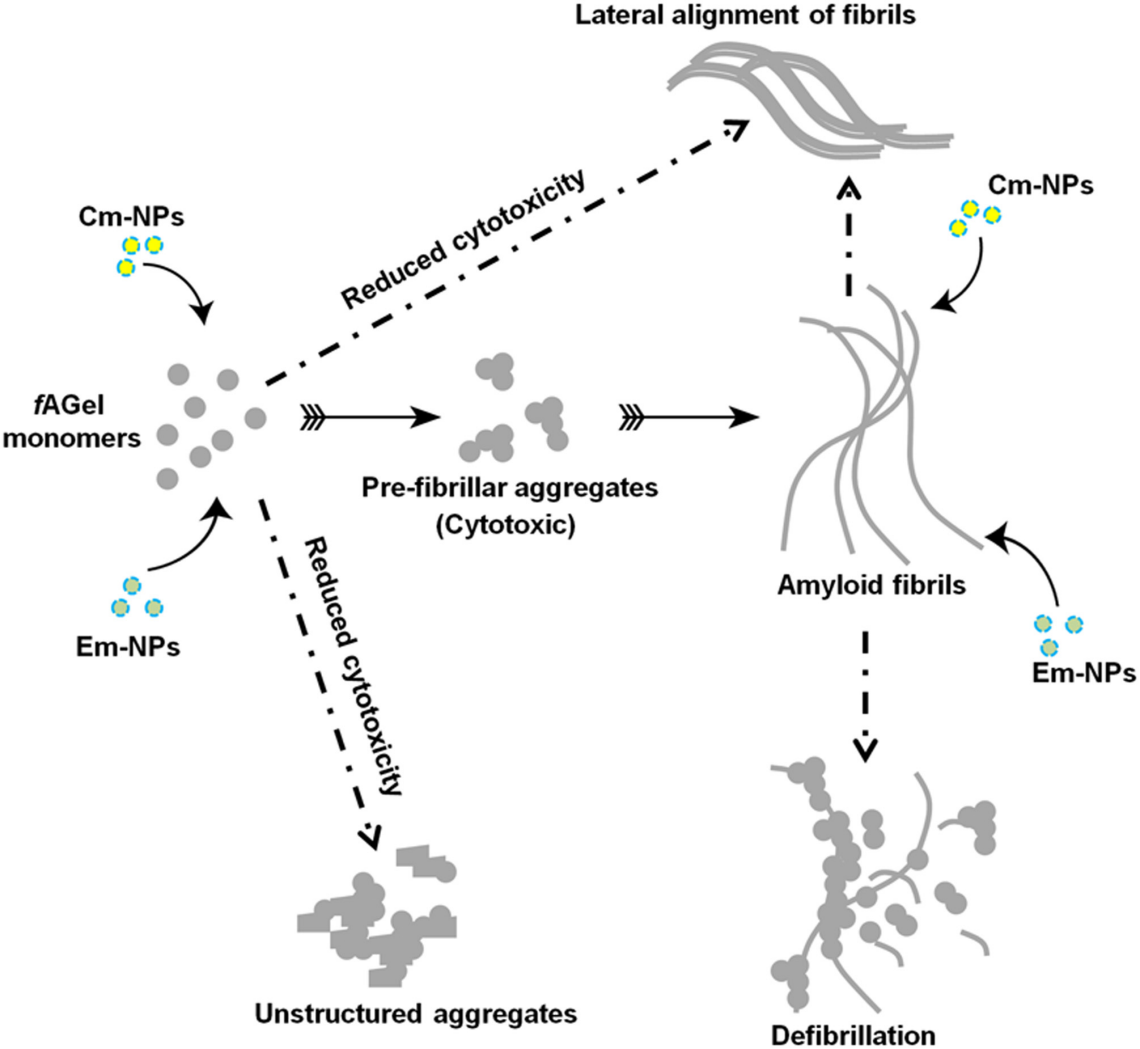
Schematic representation of differential effect of NPs on *f*AGel amyloidogenesis.

We report diametrically opposite effects of curcumin and emetine loaded PLGA NPs on the amyloid aggregation of disease-associated gelsolin fragment (*f*AGel). While Cm-NPs accelerate fibrillar aggregation in *f*AGel, Em-NPs divert the aggregation into an alternate non-amyloid state. Clearly, these categorically distinct aggregates indicate discrete set of molecular interactions between each compound-loaded NPs and *f*AGel. A complete disappearance of lag phase at higher Cm-NP concentrations suggested a nucleation-independent polymerization event, culminating into thick mature fibrils at latter stages of incubation (S5a Fig, and Fig 5C). This effect was similar to other reports on amyloid acceleration by heparin, Benzalkonium Chloride and TiO_2_ nanoparticles [54–56]. We reasoned that this is due to lateral alignment of fibrils in the presence of highly hydrophobic curcumin moieties. In the case of emetine, the irregular shaped assemblies resulted from steric blockage by the bulky isoquinolines of emetine as reported earlier, and as seen in the corresponding SEM images (S5 Fig). In each one of these cases, the amount of compound required to bring about the same effect as that of free compound is drastically reduced (Table 2). This is because PLGA nano-encapsulation enhanced the solubility of the compounds, causing several fold increase in their accessibility to *f*AGel. This results in effective modulation of the aggregation pathway even at 1000-times lower concentrations as compared to free compounds (Table 2, Fig 9). The SEM images taken after extended periods of incubation (2 weeks) displayed sustained and extended amyloid modulation induced by the two compounds (S5 Fig). In the case of PLGA-curcumin, our study clearly indicates that the amyloid-modulating effect persisted long after the in vivo absorption and elimination half lives (0.3 h and 1.7 h, respectively) of free curcumin [57]; a desirable quality for any potential drug candidate. This property is particularly useful for the effectiveness of emetine as an amyloid modulator. Emetine affects protein synthesis and thus is detrimental for cell growth at its higher concentrations [52]. PLGA encapsulation however, bypasses this drawback and enables controlled and prolonged release at drastically reduced sub-nanomolar concentrations. Another interesting aspect is that the resulting aggregates in both the cases showed reduced cytotoxicity, re-enforcing the concept that cytotoxic species in amyloid pathways are intermediates, rather than the end products [58]. In our case, both the NPs altered or bypassed the formation of these toxic intermediates and resulted in significant reduction in amyloid toxicity (Fig 9).

**Table 2:** Comparison of parameters between nano-encapsulated versus free compounds.

Parameter	Curcumin (free)	Curcumin Nanoparticle (Cm-NPs)	Emetine (free)	Emetine Nanoparticle (Em-NPs)
Solubility	Low	high	Moderate	high
Concentration*	25 μM	25.7x10^-3^μM	25 μM	28x10^-3^μM
Time^#^	10.9 h	NIL	21.4 h	34.6 h
Release	Poor	Sustained	low	Sustained

* Concentration of free curcumin and emetine used in Arya P *et al*. 2014.

^#^ Reduction of lag phase (curcumin) and increase in lag phase (emetine). Free compounds data taken from Arya P *et al*. 2014.

## Conclusion

Rational design of effective therapeutics against amyloid diseases requires a targeted approach, owing to the complex mechanism of amyloidogenesis. The disease progression arrogates biological defence mechanisms that involve multiple proteins interacting together. Biologically compatible drug delivery systems may selectively interfere at different steps of the complex protein aggregation pathway. Curcumin, a traditionally known multi-faceted Indian herb has been utilized mostly as a food supplement rather than a drug molecule. On the other hand, emetine is a plant alkaloid used as an antiprotozoal drug. In both cases, poor pharmacokinetic properties hinder their sustained usage as multi-targeted therapeutic agents. From our study, we conclude that as compared to free compounds, PLGA encapsulation not only increases the solubility of curcumin and emetine but also reduces their concentration dependence for aiding amyloid modulation (~1000 fold reduction). Although further conclusions on druggability of both these NPs depend on in *vivo* studies, our preliminary results suggest therapeutic importance of curcumin and emetine as starting molecules for designing anti-amyloidogenic drugs. As a whole, with the advantage of controlled release, the biocompatibility and biodegradability of PLGA makes our conjugate NPs novel drug-like therapeutics against amyloid toxicity.

## Supporting Information

S1 Fig Charge distribution and particle size distribution profiles of Cm-NPs (a) & (b); Em-NPs (c) and (d); PLGA-NPs (e) and (f). (TIF)

S2 Fig Width and height analysis of *f*AGel amyloid fibers formed after 60 h incubation. (TIF)

S3 Fig Width and height analysis of *f*AGel fibrillar aggregates formed after 60 h incubation with Cm-NPs (0.1 mg/ml). (TIF)

S4 Fig Width and height analysis of *f*AGel unstructured aggregates formed after 60 h incubation with Em-NPs (0.1 mg/ml). (TIF)

S5 Fig Scanning electron micrographs of (a) *f*AGel incubated with Cm-NPs and (b) *f*AGel incubated with Em-NPs. The images were captured after two weeks of incubation with 0.1 mg/ml of respective NPs. The scale bars represent 100 μm.
(TIF)
