# Supplementary figures and images for: Gelsolin Amyloidogenesis Is Effectively Modulated by Curcumin and Emetine Conjugated PLGA Nanoparticles
Source: PLoS One. 2015 May 21;10(5):e0127011. doi: 10.1371/journal.pone.0127011 (PMC4440822; doi:10.1371/journal.pone.0127011)

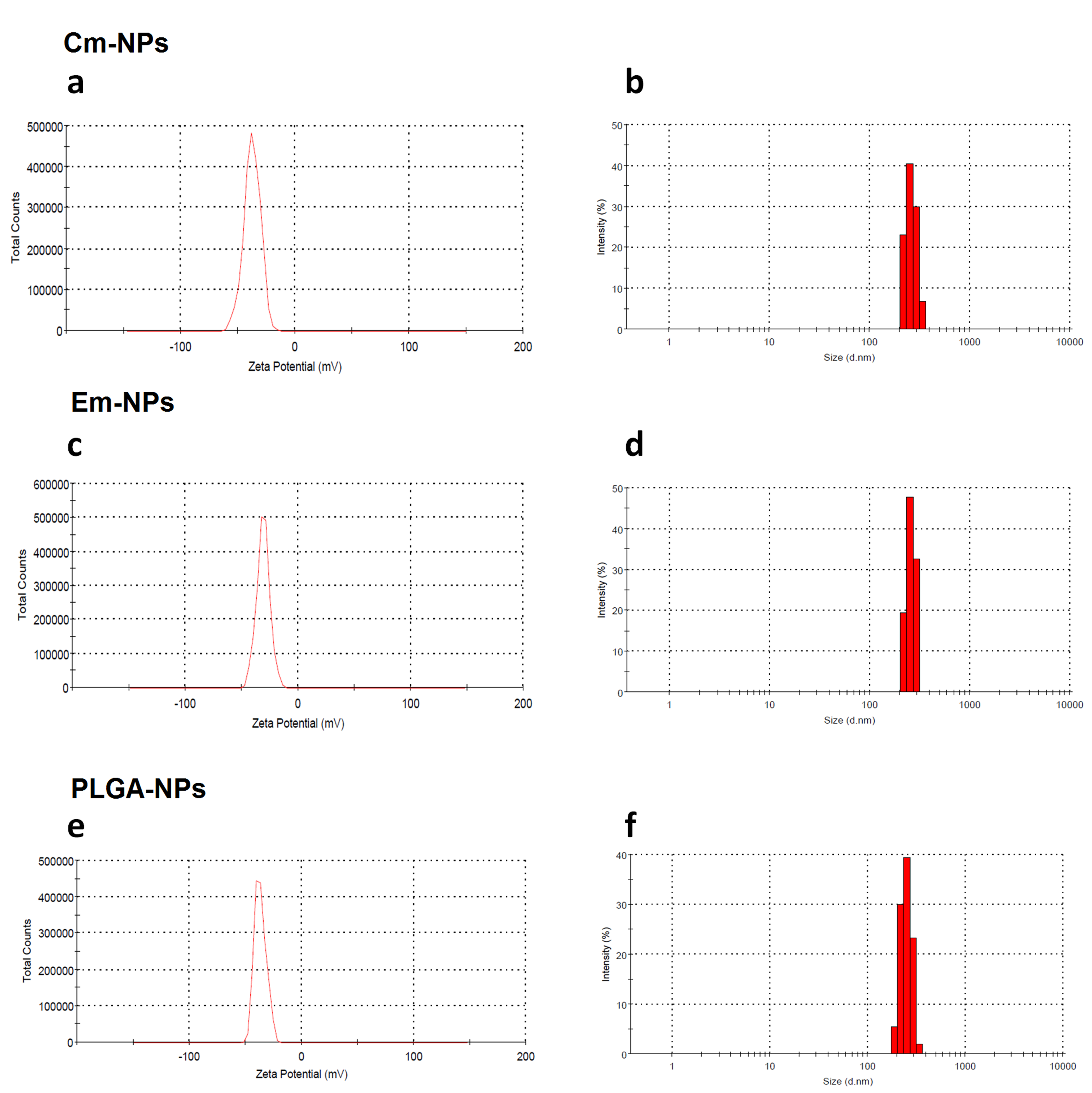

Supplement: S1 Fig — (TIF) [file pone.0127011.s001.tif]

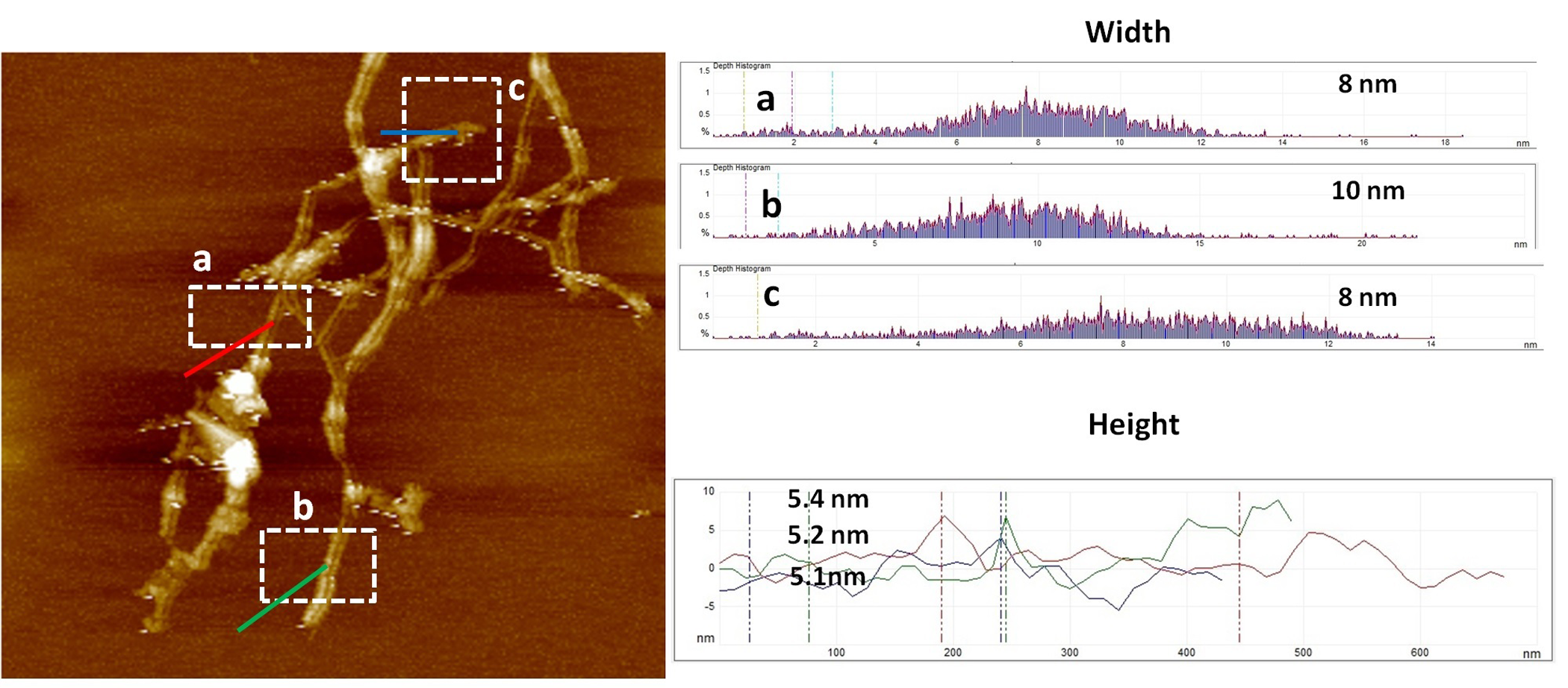

Supplement: S2 Fig — (TIF) [file pone.0127011.s002.tif]

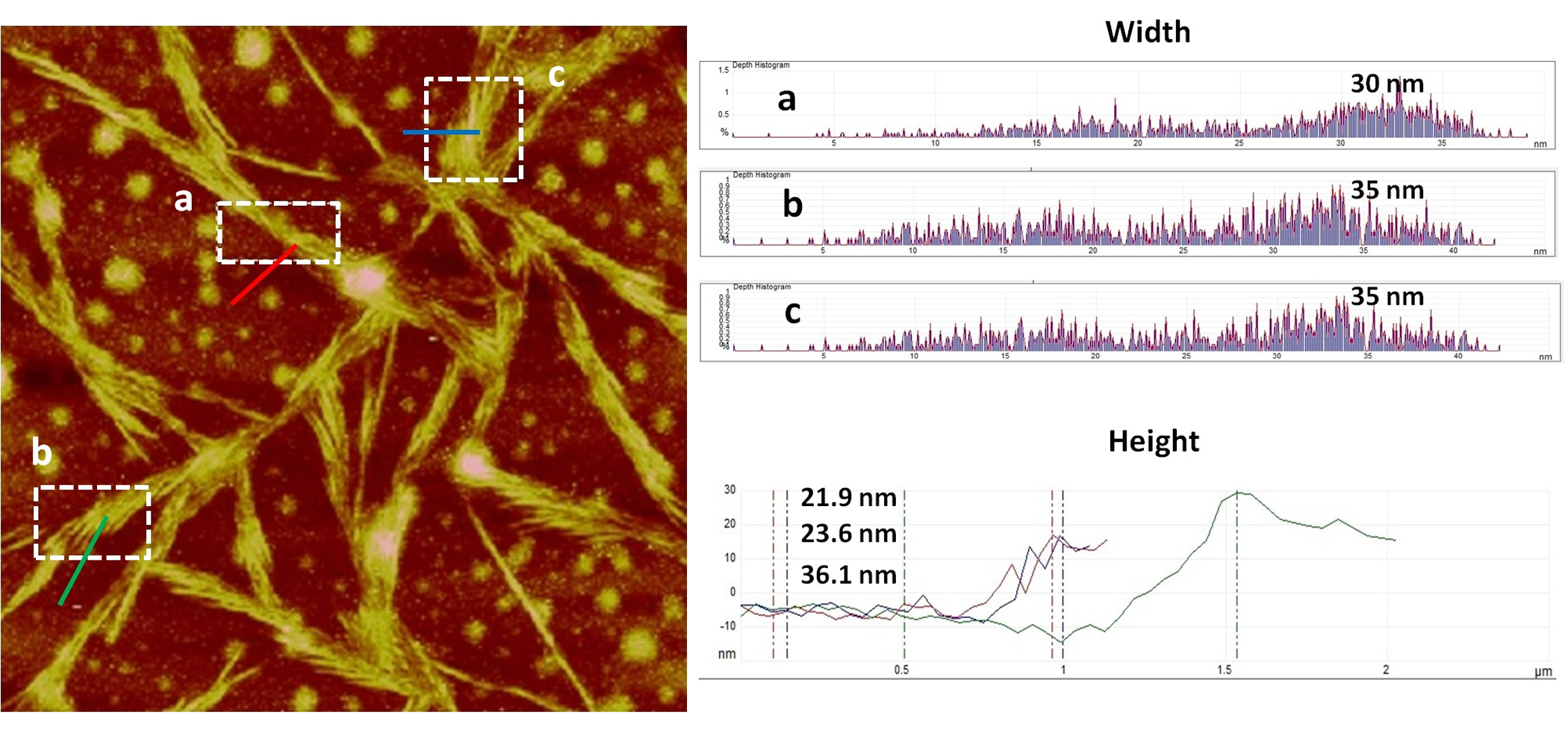

Supplement: S3 Fig — (TIF) [file pone.0127011.s003.tif]

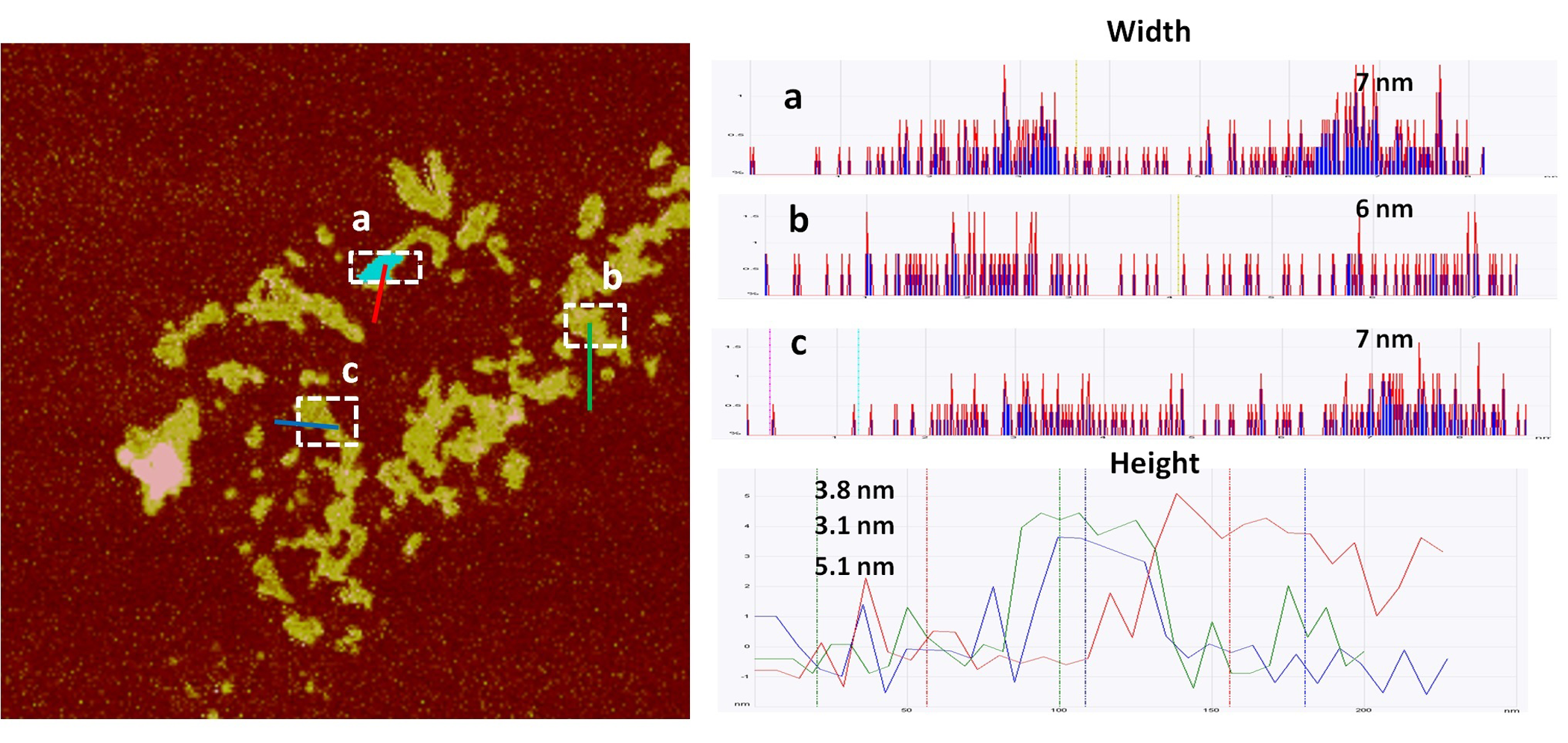

Supplement: S4 Fig — (TIF) [file pone.0127011.s004.tif]

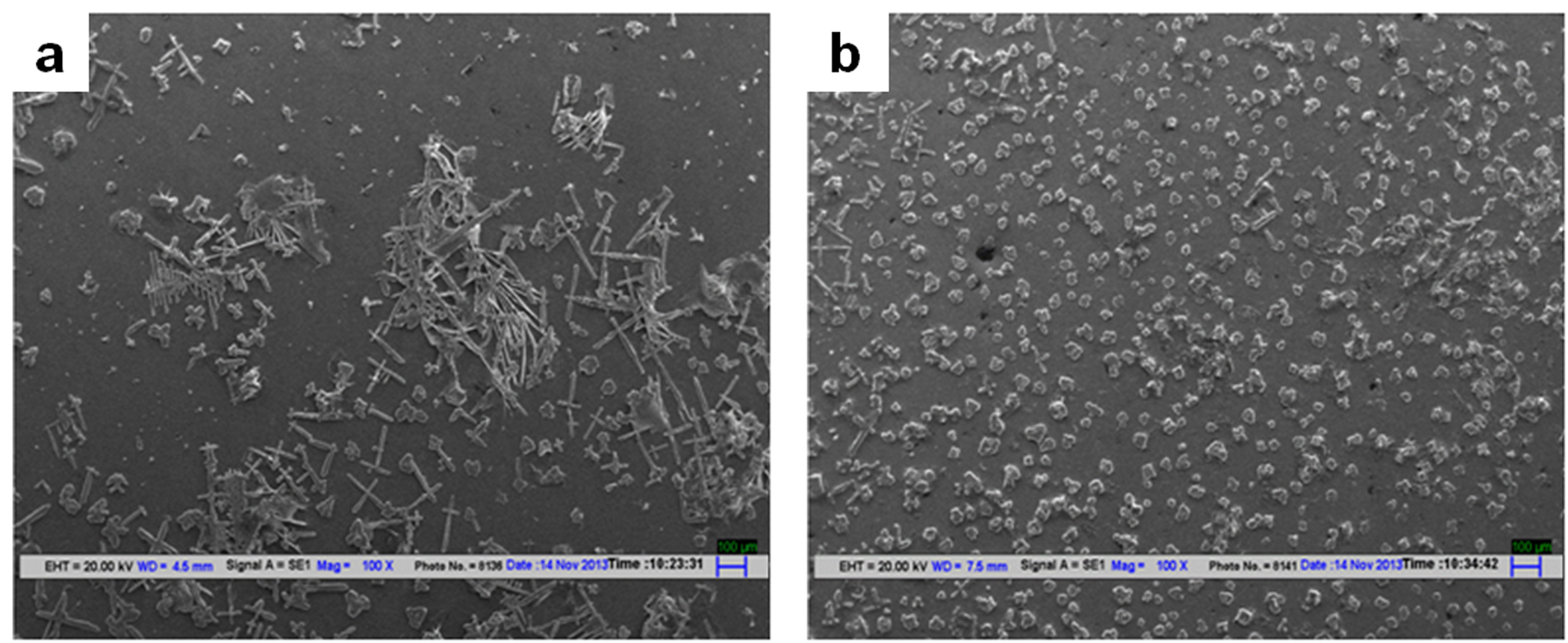

Supplement: S5 Fig — The images were captured after two weeks of incubation with 0.1 mg/ml of respective NPs. The scale bars represent 100 μm. (TIF) [file pone.0127011.s005.tif]
